# Trick or twit?

**DOI:** 10.15252/embr.202256599

**Published:** 2022-12-14

**Authors:** Howy Jacobs

**Affiliations:** ^1^ Tampere University Tampere Finland; ^2^ La Trobe University Melbourne VIC Australia

**Keywords:** History & Philosophy of Science, Science Policy & Publishing

## Abstract

How can we turn the tide of willful ignorance about scientific issues in the age of social media?

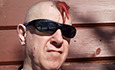

Conspiracy theorists nowadays have free rein on social media to propagate untruths and distortions. And science is a frequent target, especially where experimentally verifiable data contradicts long‐held beliefs or seems opposed to a deeply rooted ideology. But, even if science follows no ideology other than a fundamental commitment to truth, ensuring that truth prevails is an enormous task in which we frequently appear to be failing. One ill‐informed comment on a social media platform from an ideologically driven influencer can outweigh the view of thousands of scientists based on reliable findings well‐documented in the scientific literature.

How can we begin to turn back this tide? The answer most commonly given is that we need to engage much more with the general public and address the most contentious issues: climate change, vaccines, animal experiments, the environmental benefits of GMO crops, and so on. Like many others, I have argued that our research funders and universities should not only require us to commit to public engagement of this kind, but should also fund us properly to do so, and actually measure the impact of our so doing. All of us need to be able to communicate the very basic aspects of what we study and why we think it is important, because nobody else is going to do it for us. And, since this type of communication needs to be learned and perfected, we should all receive proper training at an early stage in our academic careers. At present, like much else in our profession, we are expected to just make up these skills “on the job.”

Much of the effort in informing at least the coming generation about the above issues and many others has to come through high schools, where students are still exposed to a wide portfolio of subjects, in contrast to the specializations of college and university studies. But it's a huge task. Making an annual visit to each of many local schools involves a large commitment of time, and is always a learning curve: learning how students react and respond and tuning in to their concerns are as important as the actual facts we seek to impart. But even this would not be enough.

However beneficial such efforts may be in informing the public and in winning the relevant arguments, it does not reach those who do not wish to be informed, or who are impervious to any information we would seek to impart. They tend to believe whatever social media influencers tell them, because they automatically distrust anyone perceived as part of the societal elite, which includes us scientists. In the eyes of a disaffected teenager, being smart is often derided; the most successful teachers are the ones who can meet their students' expectations and make them feel comfortable in raising their gaze. Again, it's an art in which many of us are not schooled at all. We need to earn the respect of our audience, not just try to impress them with stuff they cannot follow or they do not care about. Otherwise, we are just behaving as the despised elite and reinforcing the prejudices we seek to counteract. Knowledge is meaningless if it is not successfully shared.

Of course, the main task of teaching what science is and how it can be judged must be left to teachers, even though our presence can validate what is taught. And it does not stop with teaching the “facts” of science. It must begin with something much more fundamental: the scientific method, how one goes about planning an experiment and analyzing the results, and the ways in which scientific findings are communicated, disseminated, challenged, and reconfirmed. Obviously we cannot expect high school students or the general public to start reading the scientific literature. Most of us can barely keep up with it in our own narrow field. What we need to do, somehow, is impart an understanding of what the scientific literature is and what it contains, so as to lay the groundwork for more public trust in science. How are findings reported? How are they validated and by whom? How do we ensure that data is presented and interpreted in an unbiased way? How are errors and misinterpretations corrected? All this should be part of basic science education, not just teaching about the geological record, the periodic table or the components of the cell.

Teachers must be prepared not only to say how all the above is done or should be done, but also explain what mechanisms exist for identifying and preventing potential bias. How do we make sure that peer‐review is not simply the elite validating its own prejudices? Where is democratic control and accountability—and should there even be any in science? What did it mean when scientific articles were marked as “advertisement” and why is this formula no longer in use? What does it mean when authors declare “no conflict of interest” and who checks this? If someone is paying for their work to be published, does that mean it is inherently untrustworthy? And who gets to decide what experiments are to be done and funded?

These are questions that we all grapple with in professional life, especially when it comes to reporting our own findings, reading and evaluating the work of others, satisfying editors and peer‐reviewers, getting our work noticed sufficiently to warrant an invitation to present at an international conference, reporting to funders and to faculty committees dealing with tenure and promotion, or just getting the funding and the job or fellowship in the first place. But if science is to stem the growing tide of disinformation and propaganda, our community has to persuade the rest of society that its findings are valid, explain why that is so, and demonstrate what safeguards are in place to ensure this. Thus, we—and all the teachers who do the heavy lifting—need to explain not just the content of what we publish, but the universally applied criteria for validating our findings—controls, statistics, replication—and the mechanisms behind publication.

And I would go even further. If these practices that define how we conduct ourselves as professional scientists cannot be explained and justified to a class of inquisitive 15‐year‐olds, then they must be changed.

## Disclosure and competing interests statement

The author declares that he has no conflict of interest.

